# Roadmap to Innovation: Charting California’s Path to Strengthening Caregiving Services and Supports

**DOI:** 10.1093/geroni/igaf122.995

**Published:** 2025-12-31

**Authors:** Rachael Fulp-Cooke, Heather Young

## Abstract

The landscape of family caregiving is evolving, shaped by demographics and changing care needs. In California, nearly seven million family caregivers form the foundation of the state’s Long-Term Services and Supports (LTSS) system, enabling older adults and people with disabilities to remain in their homes and communities. As the population ages, and more individuals live with chronic conditions or functional and cognitive limitations, the demand for caregiving is increasing while the number of available caregivers is declining. Caregivers span all ages, and represent diverse backgrounds, experiences, and identities. While caregivers share common challenges, factors such as income, language, and geographic location shape their caregiving experience in distinct ways with significant policy implications. The California Department of Aging (CDA) partnered with the Family Caregiving Institute (FCI) at the UC Davis Betty Irene Moore School of Nursing to develop the California Caregiver Equity Roadmap (“Roadmap”). Launched in July 2023, this initiative aims to strengthen publicly funded caregiver services and supports advancing the fourth goal of the California Master Plan for Aging: Caregiving That Works. This project also assessed how California’s caregiving efforts align with the 2022 National Strategy to Support Family Caregivers and identified promising practices from other states. This symposium will explore the Roadmap’s development, including the community engagement process and data analyses that shaped it. Additionally, presenters will discuss caregiving strengths and opportunities in California and examine innovative caregiving initiatives nationwide to inform efforts to strengthen caregiver services and supports. Family Caregiving Interest Group Sponsored Symposium

